# The Effects of Percutaneous Mitral Balloon Valvuloplasty on the Left Atrial Appendage Function in Patients With Sinus Rhythm and Atrial Fibrillation

**DOI:** 10.15171/jcvtr.2014.07

**Published:** 2015-03-29

**Authors:** Naser Aslanabadi, Iraj Jafaripour, Mehrnoush Toufan, Bahram Sohrabi, Ahmad Separham, Reza Madadi, Hossein Feazpour, Yosef Asgharzadeh, Mostafa Ahmadi, Abdolrasol Safaiyan, Samad Ghafari

**Affiliations:** ^1^ Cardiovascular Research Center, Tabriz University of Medical Sciences, Tabriz, Iran; ^2^ Department of Biostatistics, Faculty Health, Tabriz University of Medical Science, Tabriz, Iran

**Keywords:** Balloon Valvuloplasty, Atrial Appendage, Atrial Function

## Abstract

***Introduction:*** Mitral stenosis (MS) causes structural and functional abnormalities of the left atrium (LA) and left atrial appendage (LAA), and studies show that LAA performance improves within a short time after percutaneous transvenous mitral commissurotomy (PTMC). This study aimed to investigate the effects of PTMC on left atrial function by transesophageal echocardiography (TEE).

***Methods:*** We enrolled 56 patients with severe mitral stenosis (valve area less than 1.5 CM^2^). All participants underwent mitral valvuloplasty; they also underwent transesophageal echocardiography before and at least one month after PTMC.

***Results:*** Underlying heart rhythm was sinus rhythm (SR) in 28 patients and atrial fibrillation (AF) in remainder 28 cases. There was no significant change in the left ventricular ejection fraction (LVEF), left ventricular end diastolic dimension (LVEDD), or the left ventricular end systolic dimension (LVESD) before and after PTMC in both groups. However, both groups showed a significant decrease in the left atrial volume index (LAVI) following PTMC (P=0.032 in SR and P=0.015 in AF group). LAA ejection fraction (LAAEF) and the LAA emptying velocity (LAAEV) were improved significantly after PTMC in both groups with SR and AF (P<0.001 for both).

***Conclusion:*** Percutaneous transvenous mitral commissurotomy improves left atrial appendage function in patients with mitral stenosis irrespective of the underlying heart rhythm.

## Introduction

Rheumatic mitral valve stenosis (MS) is a relatively common cause of valvular disease in developing countries such as Iran.^1^ MS is known to cause structural and functional abnormalities of the left atrium (LA) and left atrial appendage (LAA). Developmentally, the LAA is a remnant of the embryonic atrium, whereas the smooth left atrial cavity comes from an outgrowth of the pulmonary veins.^2^ Importantly, the LAA has an important pathophysiological function because it is more compliant than the LA and its contractile capacity prevents blood stasis.^3,4^

Recent studies have also shown a beneficial role of the LAA in modulating left atrial pressure.^5^ Impaired function of the LAA, such as reduced flow velocity, typically results in LA thrombus formation,^6^ with LAA thrombi more prevalent in presence of low velocities (<20 cm/s) than higher velocities (17% vs 5%, respectively).^7^ LAA velocity is an important predictor of thrombus formation, independent of other hemostatic variables, including platelet and thrombotic activity.^8^

The risk of cerebrovascular accident (CVA) increases in patients with rheumatic atrial fibrillation (AF) by 6- to 17-fold,^9^ and the disturbed LAA and LA function in patients with MS in sinus rhythm (SR) can also increase the risk of CVA.^10^ Indeed, mitral stenosis causes resistance against active and passive LA and LAA depletion, chronic pressure and volume overload, and LA myopathy. Eventually, it causes slow blood flow and blood stasis in the LAA and thrombus formation.^11^

Studies show that LAA performance improves within a short time after percutaneous transvenous mitral commissurotomy (PTMC) in patients with AF or in SR.^12,13^ Recovery of LAA velocity after PTMC depends directly upon improving the mitral valve hemodynamics and reducing the trans-mitral gradient.^12^ Since its introduction in 1984 by Inoue et al, PTMC has become established as a safe and effective treatment for rheumatic MS, with results that are equivalent to surgical valvotomy.^14^ In addition, transesophageal echocardiography (TEE) is a high-resolution imaging technique that can be used to evaluate the performance of LAA. This study aimed to clarify the effects of PTMC on left atrial function by TEE.

## Materials and methods

We included 67 patients with severe MS (valve area less than 1.5 cm²) referred to Madani Heart Center for PTMC between September 2013 and July 2014. Patients with the following criteria were excluded from the study: mitral regurgitation > 2+, LA and LAA thrombus, hypertension, diabetes mellitus, ischemic heart disease, LVEF < 35%, history of myocardial infarction, New York Heart Association (NYHA) functional class IV, and patients who did not consent to TEE after PTMC. After the study, patients with a mitral valve area less than 1.5 cm² and mitral regurgitation > 2+ were also excluded. Ethics committee evaluated the research plan and informed consent were obtained at the time of enrollment.

Of the 67 included patients, 3 with LA thrombus, 3 with mitral regurgitation > 2+, 1 with diabetes mellitus, and 2 with ischemic heart disease were excluded. Another 2 patients were excluded because they removed their consent after the procedure. Therefore, the final study comprised 56 patients, including 28 patients in SR and 28 patients with AF. All patients underwent mitral valvuloplasty by the Inoue method.

**Figure 1:**
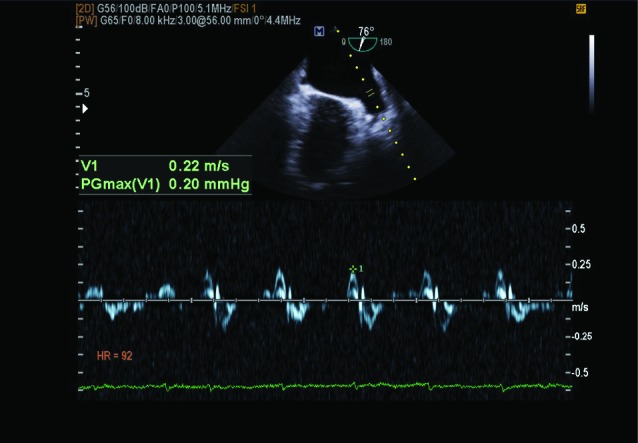
left atrial appendage velocity.

TEE was performed before PTMC and at least one month after. Patients fasted for at least 4 hours. After local anesthesia, Patients were placed in the lateral decubitus position. Pulse oximetry and cardiac monitoring were attached before TEE, which was performed using the Vivid 7 (GE, Norway) machine and TEE 2.5–3.5 mHz probe. With the TEE probe in the mid-esophagus at a 65–75 degree angle, the LAA was exposed. We then put the sample volume pulse wave (PW) Doppler in the mouth of the LAA, slightly toward its body, and measured the LAA velocity (Figure 1).

We measured the fractional area change as its ejection fraction (EF) by the Simpson method, tracing the end diastolic and end systolic area of the LAA. To evaluate the left atrial volume index (LAVI), we traced the LA area in 2-chamber and 4-chamber views, measured the LA depth in both views, and then calculated the LAVI according to the area length formula. To measure the MVA, we used direct planimetry in the short axis view during diastole.

The following echocardiographic data were measured according to the American Society of Echocardiography guidelines: LVEF, LAVI, left ventricular end diastolic dimension (LVEDD), left ventricular end systolic dimension (LVESD), pulmonary artery systolic pressure (PASP), and the mitral valve peak and mean gradients (MVPG and MVMG, respectively).

To ensure the accuracy of the echocardiographic parameters, all echocardiographic examinations were performed by an echocardiography specialist and we used mean values obtained from three consecutive beats.

### Statistical methods

Before intervention, the important and influential variables were adjusted between the SR and AF groups. Statistical evaluation was performed using IBM SPSS Statistics for Windows, Version 22.0 (IBM Corp., Armonk, NY, USA). The level of significance was set at 0.05, and all results were expressed as means ± standard error of the mean (SEM).

## Results

Table 1 summarizes the baseline characteristics of the patient population**.** Of the 56 participants, 28 were in SR and 28 were in AF. In the SR group, 28.6% were men and 71.4 % were women, compared to 32.1% and 67.9%, respectively, in the AF group. There was no significant difference in the mean age of the patients in SR and AF groups. The time interval between PTMC and follow-up echocardiography was comparable between the SR and AF groups. Spontaneous echo contrast (SEC) was observed in 8 and 13 patients in the SR and AF groups, which completely disappeared in 5 and 4 patients, respectively. The Wilkins echocardiographic score for the SR and AF groups was 7.1 ± 0.6 and 7.2 ± 0.7, respectively (P = 0.579).

**Table 1:** Baseline characteristics of the patient population.

Characteristics	Patients		P value
	Sinus Atrial	fibrillation	
Age	43.25±2.056	46.64±1.614	0.200
Sex
Male	8 (28.6%)	9 (32.1%)	0.771
Female	20 (71.4%)	19 (67.9%)
Height (Cm)	158.89± 1.402	158.78± 1.498	0.959
Follow up ECHO (Mon)	6.86 ± 3.923	6.64 ± 3.654	0.574
Wilkins Score	7.46 ± 0.149	7.57± 0.119	0.579

Follow up ECHO: Time interval between PTMC and follow up echocardiography

In the SR group, the NYHA functional class before PTMC was class III in 24 patients and class II in 4 patients. After PTMC, 26 patients were in class I and 2 patients were in class II (P<0.001). In the AF group, 25 patients were in class III and 3 patients were in class II before PTMC, which improved to class I for 21 patients and class II for 7 patients after PTMC.

### Echocardiographic findings

Table 2 shows the comparison of TEE variables before and after PTMC. In the SR group, LVEF (p = 0.324), LVEDD (P = 0.479) and LVESD (P = 0.367) did not change significantly after PTMC. However, LAVI (P = 0. 032), MVPG (P<0.001), MVMG (P<0.001), and PASP (P<0.001) decreased significantly after PTMC with significant increase in MVA (P<0.001).

**Table 2:** TEE variables before and after PTMC in patient whit SR

Variables	Before PTMC	After PTMC	p- value
LVEF (%)	53.71 ± 0.851	53.57 ± 0.723	0. 837
LVEDD (cm)	4.611 ± 0.962	4.658 ± 0.927	0. 243
LVESD (cm)	3.303 ± 0.101	3.323 ± 0.923	0. 772
LA VI (cm^3^/m^2^)	86.696 ± 6.939	83.592 ± 6.964	0. 032
MVA plan (cm^2^)	0.90 ± 0.030	1.72 ± 0.030	<0.001
MVPG (mm Hg)	21.76 ± 1.152	10.76 ± 0.499	<0.001
MVMG(mm Hg)	8.87 ± 0.658	4.04 ± 0.256	<0.001
PASP(mm Hg)	55.785 ±1.305	39.428 ±1.282	<0.001

LVEF, Left ventricular ejection fraction; LVEDD, Left ventricular end diastolic dimension (mm);LVESD, Left ventricular end systolic dimension (mm); LAVI, left atrial volume index; MVA plan, planimetric mitral valve area; MVMG, transmitral valve mean gradient; MVPG, transmitral valve peak gradient; PASP, pulmonary artery systolic pressure; PTMC, percutaneous transvenous mitral commissurotomy SR, sinus rhytm; TEE, transesophageal echocardiography.

The results were comparable for the AF group (Table 3) in which LVEF (P = 0.367), LVEDD (P = 0.479) and LVESD (P = 0.324) did not change significantly after PTMC. Again, LAVI (P = 0.015), MVPG (P<0.001), MVMG (P<0.001), and PASP (P<0.001) decreased significantly after PTMC with significant increase in MVA (P<0.001).

**Table 3:** TEE variables before and after PTMC in patient whit AF

Variables	Before PTMC	After PTMC	p-value
LVEF (%)	53.68 ± 0.847	52.93 ± 0.672	0.367
LVEDD (cm)	4.360 ± 0.083	4.523 ± 0.0715	0.479
LVESD (cm)	3.1375 ± 0.069	3.235 ± 0.656	0.324
LA VI (cm^3^/m^2^)	82.624 ± 6.376	75.050 ± 4.188	0.015
MVA plan (cm^2^)	0.88 ± 0.025	1.65 ± 0.031	<0.001
MVPG (mm Hg)	21.10 ± 1.249	11.01 ± 0.443	<0.001
MVMG (mm Hg)	8.37 ± 0.822	3.65 ± 0.245	<0.001
PASP (mmHg)	57.68 ± 1.02	40.82 ± 0.083	<0.001

AF, atrial fibrillation; LVEF, Left ventricular ejection fraction; LVEDD, Left ventricular end diastolic dimension (mm);LVESD, Left ventricular end systolic dimension (mm); LAVI, left atrial volume index; MVA plan, planimetric mitral valve area; MVMG, transmitral valve mean gradient; MVPG, transmitral valve peak gradient; PASP, pulmonary artery systolic pressure; PTMC, percutaneous transvenous mitral commissurotomy; SR, sinus rhytm TEE, transesophageal echocardiography.

In terms of LAA performance in the SR group (Table 4), LAA end diastolic volume (LAAEDV) did not change significantly after PTMC (P = 0. 243), while LAA end systolic volume was decreased significantly after PTMC (P<0.001). However, the LAA ejection fraction (LAAEF) and LAA emptying velocity (LAAEV) were increased significantly (P<0.001 for both). Similar changes were recorded in patients with AF rhythm (Table 5).

**Table 4:** LAA Function Before and After PTMC in patient whit SR

sinus	Before PTMC	After PTMC	p-value
LAAEDV(cm^2^)	5.036 ± 0.239	4.622 ± 0.200	<0.001
LAAEDV(cm^2^)	5.036 ± 0.239	4.622 ± 0.200	<0.001
LAAEF (%)	42.60 ± 1.913	56.11 ± 2.519	<0.001
LAAEV(cm/sec)	30.26 ± 2.182	40.36 ± 2.723	<0.001

LAAEDV, Left Atrial Appendage end diastolic volume; LAAEDV, Left Atrial Appendage end systolic volume; LAAEF, Left Atrial Appendage ejection fraction ; LAAEV, Left Atrial Appendage Emptying Velocity; PTMC, Percutaneous Transvenous Mitral Commissurotomy; SR, sinus rhytm; TEE, transesophageal echocardiography.

**Table 5:** LAA Function Before and After PTMC in patient whit AF

Variables	Atrial fibrillation		p-value
	Before PTMC	After PTMC	
LAAEDV(cm^2^)	4.277 ± 0.145	3.911 ± 0.150	<0.001
LAAESV(cm^2^)	2.664 ± 0.128	2.137 ± 0.140	<0.001
LAAEF (%)	35.71 ± 1.474	38.52 ± 1.347	<0.001
LAAEV(cm/sec)	24.10 ± 1.638	26.71 ± 1.738	<0.001

AF, atrial fibrillation; LAAEDV, Left Atrial Appendage end diastolic volume; LAAEDV, Left Atrial Appendage end systolic volume; LAAEF, Left Atrial Appendage ejection fraction ; LAAEV, Left Atrial Appendage Emptying Velocity; PTMC, Percutaneous Transvenous Mitral Commissurotomy.

We also found persistent iatrogenic atrial septal defects (ASD) in 13 patients (23.2%) at follow-up TEE, but none of them were associated with a significant left to right shunt.

## Discussion

In MS, chronic tension in the LA and LAA from volume and pressure overload results in electrophysiological and electro-anatomical changes.^15^ PTMC can improve these changes by rapid decline in left atrial afterload. In this study, we found a significant reduction in MVPG and MVMG after PTMC in both the SR and AF groups that were associated with a change in patient’s clinical status from higher NYHA classes to lower ones. Our study also showed significant decrease in LAVI, LAAEDV and LAAESV after PTMC in both groups. These changes may be due to the reduction in LA afterload.

LAA velocity is an important parameter that worsens in mitral stenosis. Tenekecioglu et al.^16^ reported that the LAA pulsed-Doppler emptying velocity was > 60 cm/s in normal healthy controls. Sahin et al.^17^ compared the LAA velocities in AF patients with MS to those in healthy controls in SR and found that LAA Doppler velocities were significantly decreased in patients with MS (peak ejection velocity 24 ± 6 cm/s vs 61 ± 16 cm/s). Those data suggest the existence of dysfunction of the LAA in the MS and reflect a disturbed function ability of that structure. In our study, the LAAEV before PTMC in both the SR and AF groups was lower than normal, and we found a significant increase in LAAEV after PTMC. This was similar to the results obtained by Karakaya et al.^18^ Tatani S.B et al.^19^ found a significant improvement of the left appendicular flow after the PTMC. In other study, Porte J.M. et al^20^ showed a marked increase in LAA peak Doppler velocity After PTMC, That this increase in peak Doppler velocity was related to the decrease or regression in left atrial spontaneous echo contrast, and correlated with the increase in mitral valve area, the decrease in transmitral pressure gradient, and the increase in cardiac index. In our study, the increase in LAAEV occurred in both groups but this increase was more dramatic in the SR group than in the AF group.

We also assessed global LAA contractile function by ejection fraction estimation, and found that this was lower in patients with MS compared with standard values available for healthy controls. LAAEF is reported to be about 70% in normal controls,^17,21,22^ while it ranges from 15% to 50% in patients with critical MS.^18,23^ In our study, the LAAEF before and after PTMC was lower than normal in both the SR and AF groups. However, we found a significant increase in the LAAEF after PTMC, which was much greater in the SR group than the AF group (13.5% vs 2.8%). In contrast to our findings, Sinan İnci, et al.^24^ showed that LAAEF did not improve following successful PTMC or during the follow-up period. Atila Bitigen et al.^25^ also observed that LAAEF did not improve completely following successful PTMC.

In MS patients, the low shear and flow-velocity condition leads to red blood cells aggregation through binding between red blood cells and plasma proteins. Thus, SEC can appear in the LA and subsequently increase LA clot formation and embolic phenomenon.^26^ In our study, we observed SEC in 8 SR patients and 13 AF patients, which completely disappeared in 5 and 4 patients, respectively. Bernstein NE et al.^27^ reported SEC in 21 of 47 patients (45%) with MS in SR, and Karakaya et al.^18^ found that SEC completely disappeared in 7 of 20 patients with MS in SR after PTMC. Vijayvergiya et al.^28^ also observed significant improvement in the grading of SEC after PTMC.

Finally, we also found persistent iatrogenic ASD in 23.2% of patients at follow-up evaluation, although they were not clinically important. The incidence of ASD after septostomy for PTMC has been reported to range from 19% to 23% at short term follow-up.^29,30^ Residual ASDs with left-to-right shunting do not appear to play an important clinical role in post-PTMC patients, unless significant left-to-right shunt is present or right-to-left shunt is created, which may lead to paradoxical embolism.^30,31^

## Limitation

We excluded patients with high Wilkins echocardiographic scores and patients with LAA clots; also our study was a single center one with limited number of patients. In addition, we provided no normal control group and failed to provide long-term follow-up.

## Conclusion

This study showed that PTMC is able to improve the clinical status, reduce SEC, and improve LAA function in patients with MS in both SR and AF rhythms. Therefore, PTMC may decrease the risk of thromboembolism by improving LA and LAA performance. However, these findings should be confirmed by further large-scale studies and long-term follow-up.

## Ethical issues

This study was approved by our local ethics committee.
